# Supplementary material for: The IFIH1-A946T risk variant promotes diabetes in a sex-dependent manner
Source: Front Immunol. 2024 Feb 29;15:1349601. doi: 10.3389/fimmu.2024.1349601 (PMC10937421; doi:10.3389/fimmu.2024.1349601)
Supplement: Supplementary file 1 [file DataSheet_1.pdf]

Supplementary Material

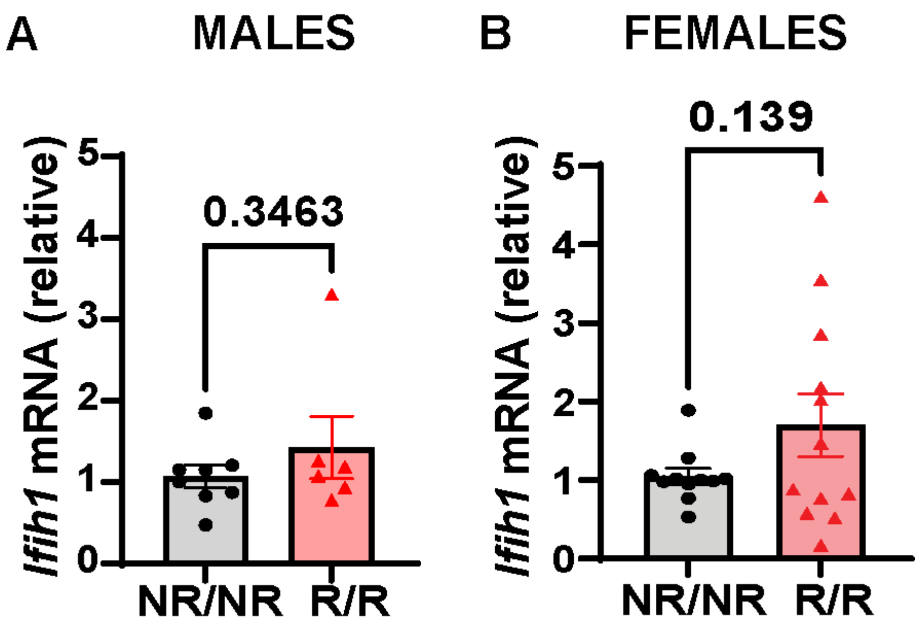

<u>Supplementary Figure 1</u>. *NOD.Ifih1*$^{R/R}$ mice do not display significant changes in *Ifih1* mRNA in 14-week-old mice. The relative mRNA expression of *Ifih1* in 14-week-old **(A)** males (*n*=9 *NOD.Ifih1*$^{NR/NR}$, *n*=6 *NOD.Ifih1*$^{R/R}$) and **(B)** females (*n*=11 *NOD.Ifih1*$^{NR/NR}$, *n*=12 *NOD.Ifih1*$^{R/R}$). P values were determined using student's unpaired *t* tests. All data are mean ± SEM.

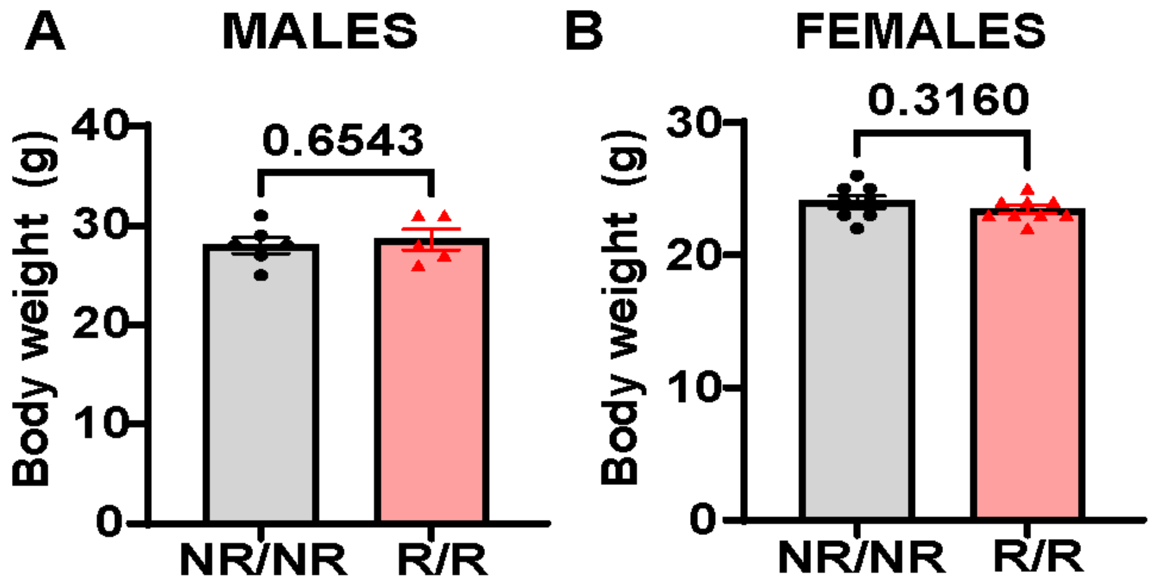

Supplementary Figure 2. ***NOD.Ifih1*$^{R/R}$ mice do not display significant changes in body weight.** Body weights measured at a single time point immediately prior to euthanasia in 14-week-old *NOD.Ifih1*$^{NR/NR}$ compared to *NOD.Ifih1*$^{R/R}$ mice in **(A)** males (*n*=6 *NOD.Ifih1*$^{NR/NR}$, *n*=5 *NOD.Ifih1*$^{R/R}$) and **(B)** females (*n*=8 *NOD.Ifih1*$^{NR/NR}$, *n*=9 *NOD.Ifih1*$^{R/R}$). P values were determined using student's unpaired *t* tests. All data are mean ± SEM.

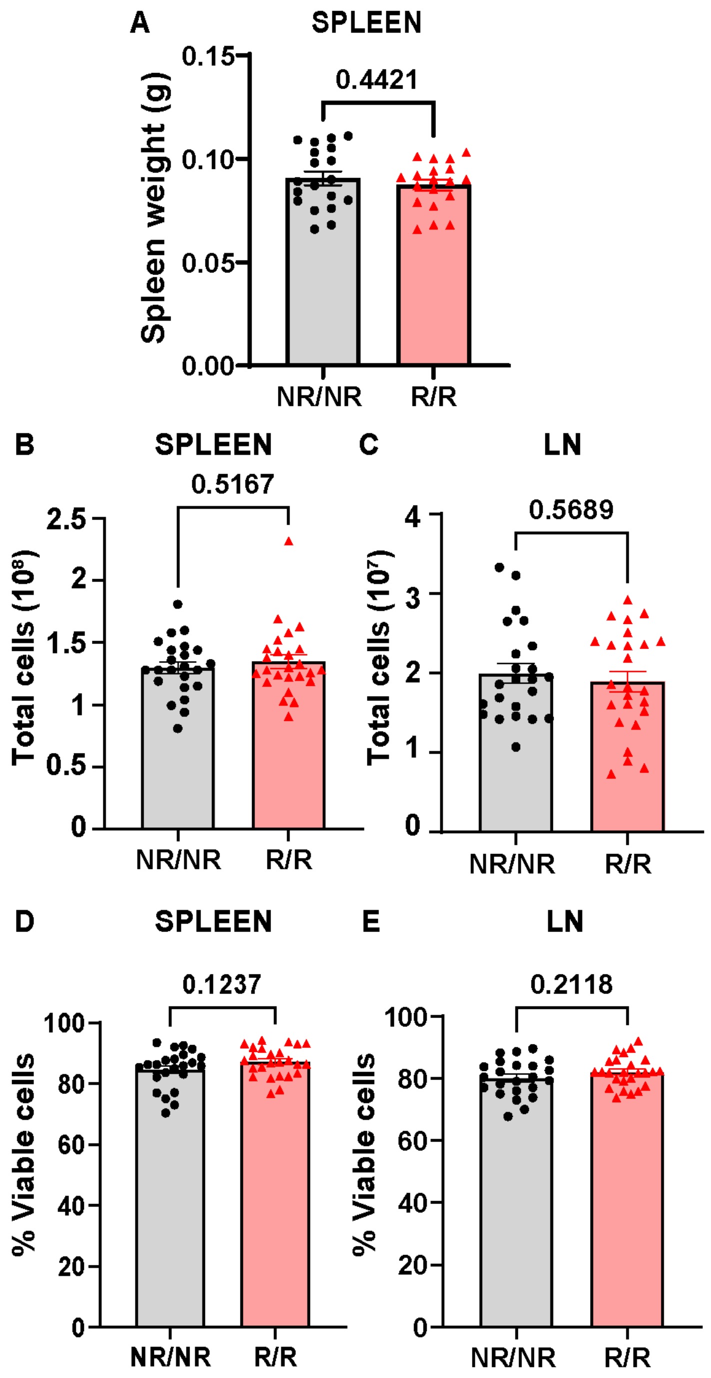

Supplementary Figure 3. ***NOD.Ifih1***$^{R/R}$ **mice do not exhibit significant changes in splenic weight or cell numbers or viability in spleens or lymph nodes. (A)** Spleen weight in grams (g) measured at the time of euthanasia in 14-week-old *NOD.Ifih1*$^{NR/NR}$ compared to *NOD.Ifih1*$^{R/R}$ mice (*n*=19 mice per genotype). **(B-C)** Total number of cells in **(B)** spleens (*n*=23 *NOD.Ifih1*$^{NR/NR}$, *n*=25 *NOD.Ifih1*$^{R/R}$) and **(C)** lymph nodes (LN, *n*=23 *NOD.Ifih1*$^{NR/NR}$, *n*=25 *NOD.Ifih1*$^{R/R}$). **(D-E)** Percent viable cells in **(D)** spleens (*n*=23 *NOD.Ifih1*$^{NR/NR}$, *n*=25 *NOD.Ifih1*$^{R/R}$) and **(E)** lymph nodes (*n*=23 *NOD.Ifih1*$^{NR/NR}$, *n*=25 *NOD.Ifih1*$^{R/R}$). P values were determined using student's unpaired *t* tests. All data are mean ± SEM.

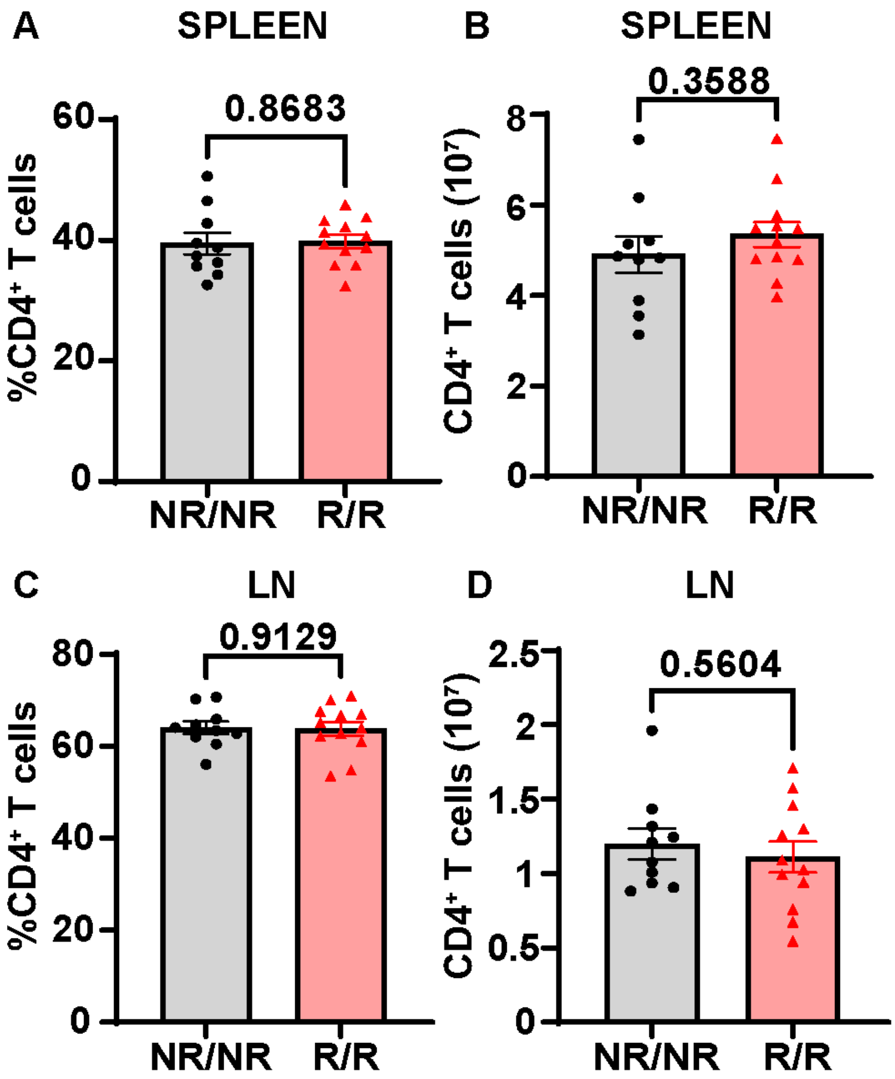

__Supplementary Figure 4__. ***NOD.Ifih1$^{R/R}$* mice do not exhibit changes in the frequency or total cell numbers of CD4^{+} T cells.** **(A-B)** CD4^{+} T cells in the spleen as **(A)** frequency of live cells and **(B)** total CD4^{+} cell numbers in *NOD.Ifih1$^{NR/NR}$* compared to *NOD.Ifih1$^{R/R}$* mice (*n*=10 *NOD.Ifih1$^{NR/NR}$*, *n*=12 *NOD.Ifih1$^{R/R}$*). **(C-D)** CD4^{+} T cells in the lymph nodes (LN) as **(C)** frequency of live cells and **(D)** total CD4^{+} cell numbers in *NOD.Ifih1$^{NR/NR}$* compared to *NOD.Ifih1$^{R/R}$* mice (*n*=10 *NOD.Ifih1$^{NR/NR}$*, *n*=12 *NOD.Ifih1$^{R/R}$*). P values were determined using student's unpaired *t* tests. All data are mean ± SEM.

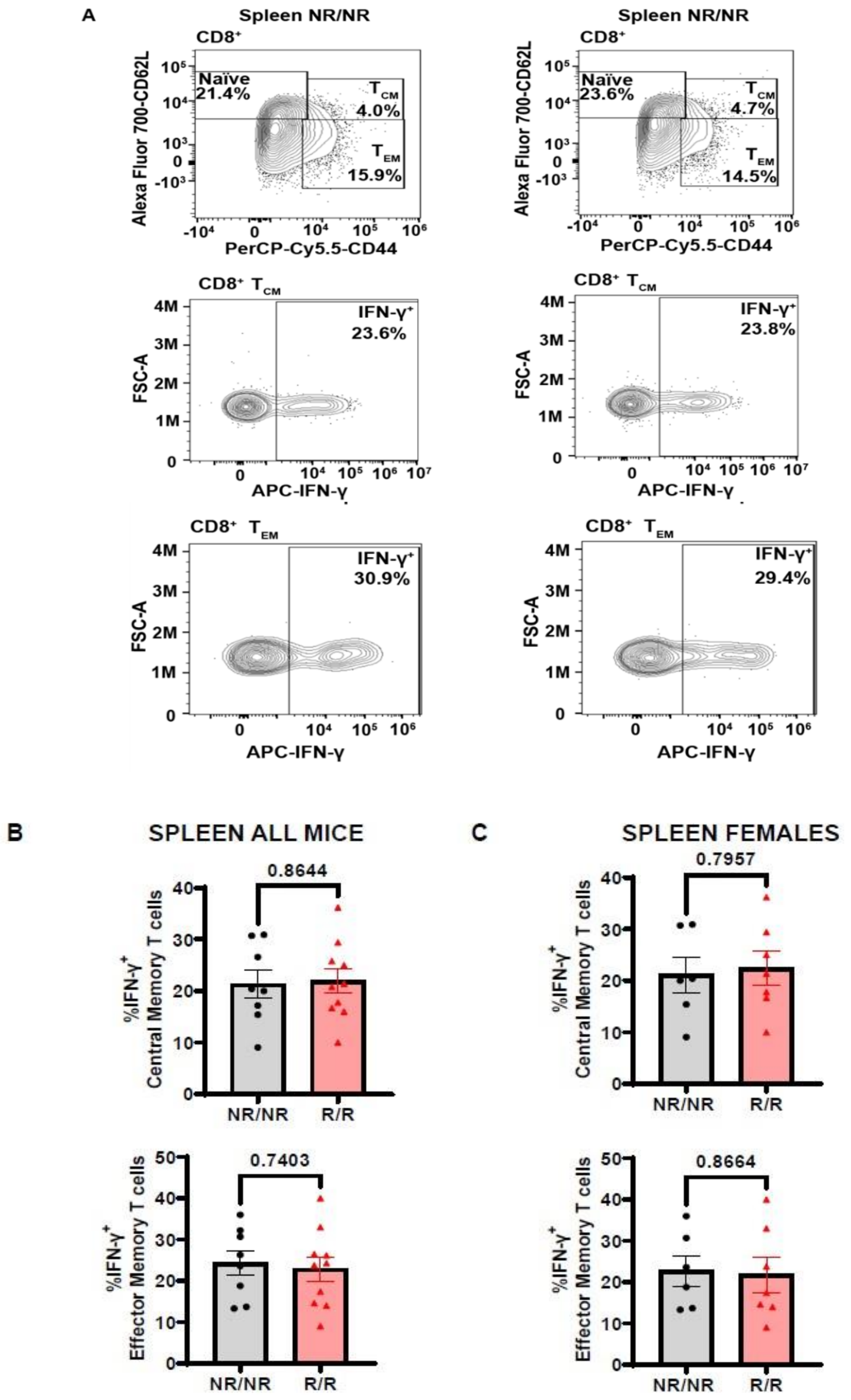

Supplementary Figure 5. ***NOD.Ifih1^{R}* does not affect the frequency of IFN-γ on splenic CD8^{+} central memory and effector memory T cells.** **(A)** Representative gating of CD8^{+} naïve (CD62L^{+}), central memory (CD62L^{+}CD44^{+}, T_{CM}), effector memory (CD62L^{-}CD44^{+}, T_{EM}), and IFN-γ^{+} CD8^{+} central memory and effector memory T cells from PMA/ionomycin-treated spleens. **(B-C)** The frequency of IFN-γ^{+} CD8^{+} central memory and effector memory T cells from PMA/ionomycin-treated lymph nodes from **(B)** all mice (*n*=8 *NOD.Ifih1$^{NR/NR}$*, *n*=10 *NOD.Ifih1$^{R/R}$*) and **(C)** females only (*n*=6 *NOD.Ifih1$^{NR/NR}$*, *n*=7 *NOD.Ifih1$^{R/R}$)*. P values were determined using student's unpaired *t* tests. All data are mean ± SEM.

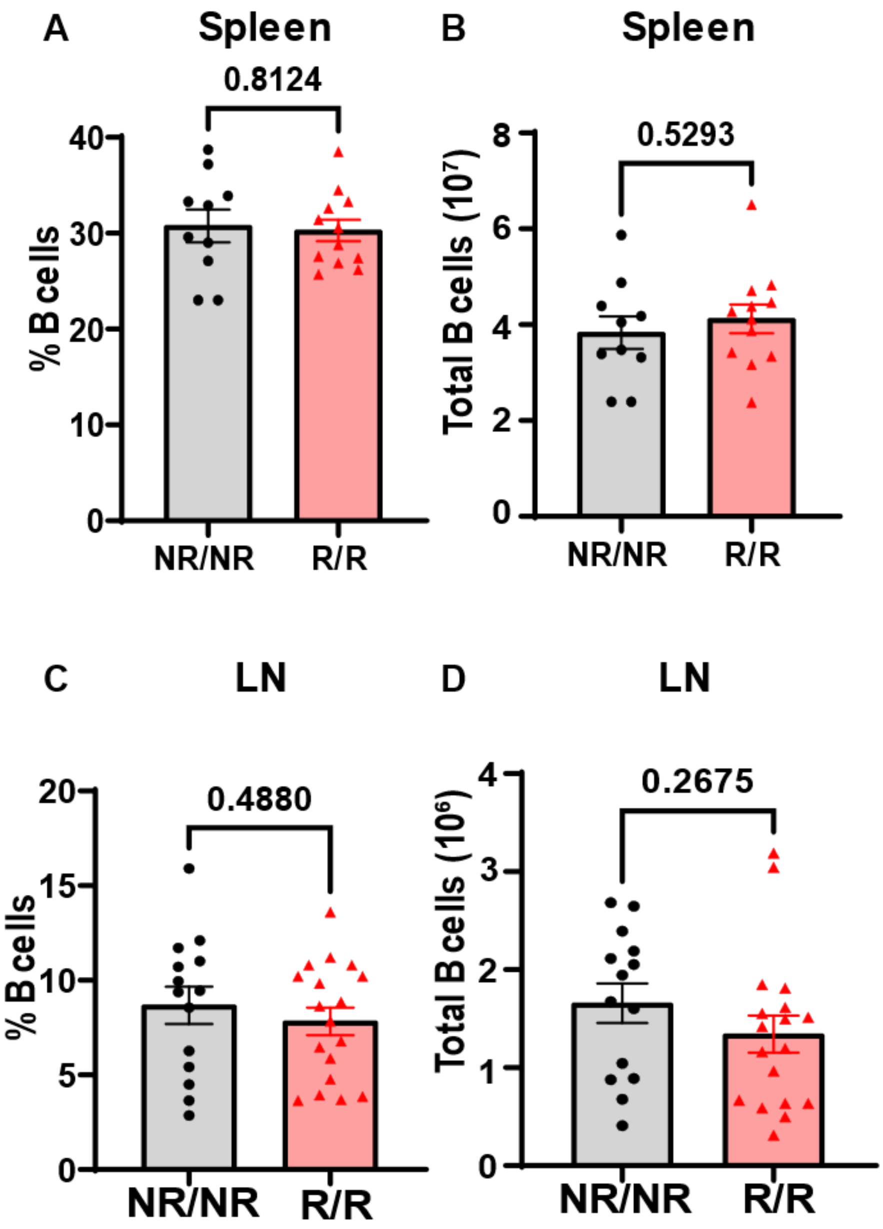

Supplementary Figure 6. ***NOD.Ifih1*^{R} does not affect the frequency or total number of B cells. (A-B)** Splenic B cells from 14-week-old mice (*n*=10 *NOD.Ifih1*$^{NR/NR}$, *n*=12 *NOD.Ifih1*$^{R/R}$) as **(A)** frequency of live cells and **(B)** total number of splenic B cells. **(C-D)** Lymph node (LN)-derived B cells from 14-week-old mice (*n*=14 *NOD.Ifih1*$^{NR/NR}$, *n*=18 *NOD.Ifih1*$^{R/R}$) as **(C)** frequency of live cells and **(D)** total number of lymph node B cells. P values were determined using student's unpaired *t* tests. All data are mean ± SEM.

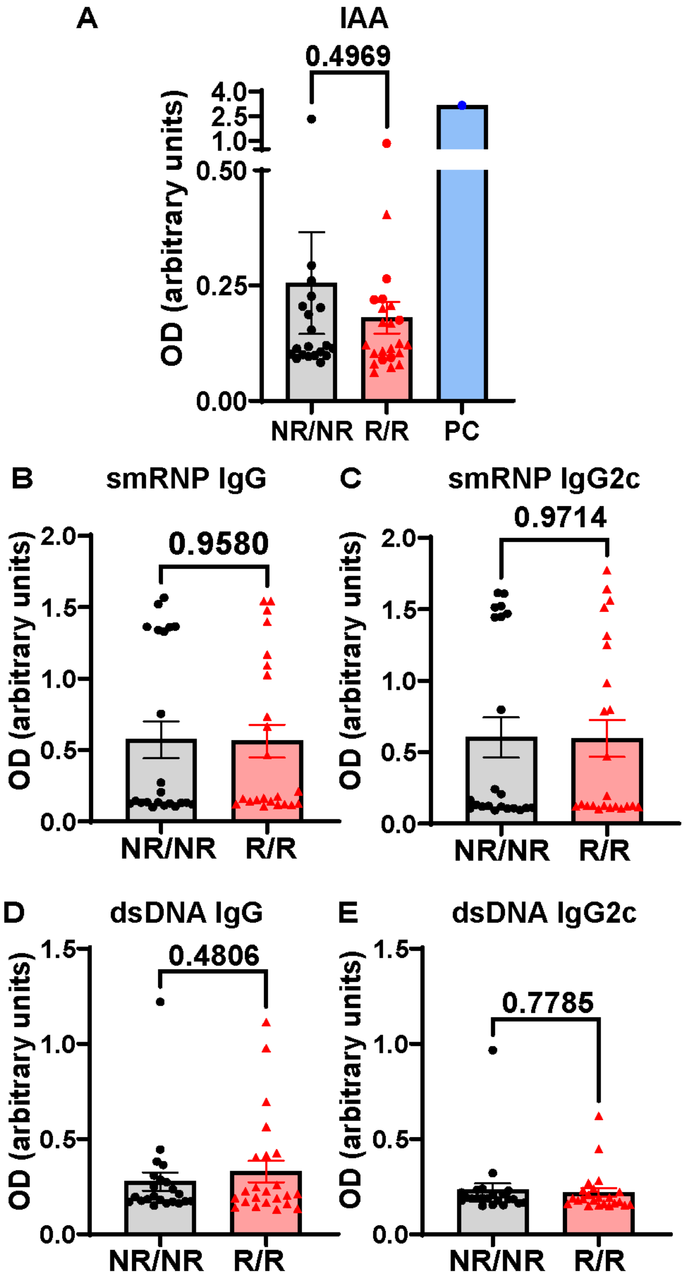

Supplementary Figure 7. *NOD.Ifih1$^{R/R}$* mice do not display significant changes in insulin, smRNP, or dsDNA autoantibody production. (A) OD measurements from insulin autoantibody (IAA) ELISAs indicative of the levels of IAA in serum from 14-week-old mice (*n*=20 *NOD.Ifih1$^{NR/NR}$*, *n*=23 *NOD.Ifih1$^{R/R}$*). PC: positive control provided by manufacturer. **(B-C)** OD measurements from smRNP **(B)** IgG and **(C)** IgG2c autoantibody ELISAs indicative of the levels of smRNP autoantibody in serum from 14-week-old mice (*n*=22 *NOD.Ifih1$^{NR/NR}$*, *n*=23 *NOD.Ifih1$^{R/R}$*). **(D-E)** OD measurements from dsDNA **(D)** IgG and **(E)** IgG2c autoantibody ELISAs indicative of the levels of dsDNA autoantibody in serum from 14-week-old mice (*n*=22 *NOD.Ifih1$^{NR/NR}$*, *n*=23 *NOD.Ifih1$^{R/R}$*). P values were determined using student's unpaired *t* tests. All data are mean ± SEM.

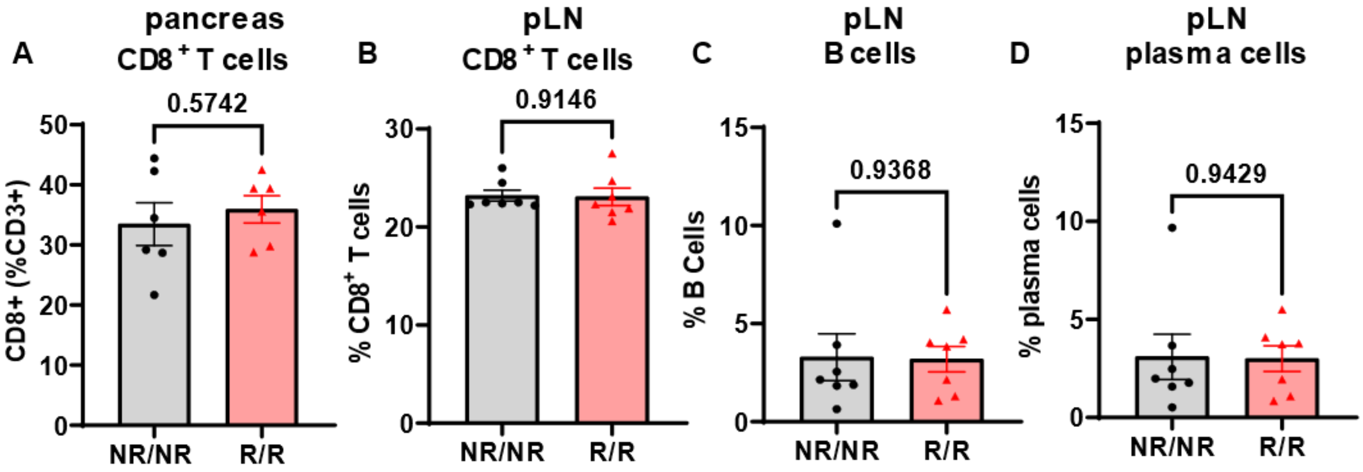

<u>Supplementary Figure 8</u>. *NOD.Ifih1*^{R} does not affect the frequency of CD8^{+} T cells in the pancreas or the frequency of immune cells in the draining lymph nodes of 14-week-old mice. (A-B) The frequency of CD8^{+} T cells in the **(A)** pancreas (*n*=6 per genotype) and **(B)** pancreatic lymph nodes (pLN, *n*=7 per genotype). **(C)** The frequency of B cells (B220^{+}) in the pancreatic lymph nodes (*n*=7 per genotype). **(D)** The frequency of plasma cells (CD19^{+}B220^{-}CD138^{+}) in the pancreatic lymph nodes (*n*=7 per genotype). All data are from 14-week-old mice. P values were determined using student's unpaired *t* tests. All data are mean ± SEM.

Supplementary Table 1. PCR and qRT-PCR Primers

Primers for PCR/sequencing	
A946 F1	5'- TTCAAGTGAGTGCAGCAAGA -3'
A946 R1	5'- AGGCAATTCCACCCACTTCT -3'
A946 R2	5'- GTGCACCATCATTGTTCCCC -3'
**Primers for qRT-PCR**	
**Gene/primer**	**Sequence**
Hprt-forward	5'-TTG CTG ACC TGC TGG ATT ACA-3'
Hprt-reverse	5'-CCC CGT TGA CTG ACT GAT CAT TAC A-3'
Ifih1-forward	5'-AGA TCA ACA CCT GTG GTA ACA CC-3'
Ifih1-reverse	5'-CTC TAG GGC CTC CAC GAA CA-3'
Ifit1-forward	5'-TGC TGA GAT GGA CTG TGA GG-3'
Ifit1-reverse	5'-CTC CAC TTT CAG AGC CTT CG-3'
Mx1-forward	5'-GGC AGA CAC CAC ATA CAA CC-3'
Mx1-reverse	5'-CCT CAG GCT AGA TGG CAA G-3'
Oas1-forward	5' CTT AGC ATG GAG CAC GGA CT-3'
Oas1-reverse	5' AGC ACC TTG GAA GCA TCT CTC-3'

Supplementary Table 2. Flow Cytometry Antibodies

Antibody	Company	Catalog #
BD Horizon™ BUV661 Rat Anti-Mouse CD19	BD Biosciences	612971
Brilliant Violet 711™ anti-mouse CD138 (Syndecan-1)	Biolegend	142519
Near IR fluorescent reactive dye	Thermo Fisher	L34994 A
Pacific Blue™ anti-mouse/human CD45R/B220	Biolegend	103227
Alexa Fluor® 700 anti-mouse/human CD45R/B220	Biolegend	103232
PE anti-mouse CD80	Biolegend	104707
PE/Cy7 anti-mouse CD3ε	Biolegend	100320
PerCP/Cyanine5.5 anti-mouse CD8a	Biolegend	100734
Brilliant Violet 711™ anti-mouse CD8	Biolegend	100759
Spark NIR™ 685 anti-mouse CD69	Biolegend	104557
Spark Violet™ 538 anti-mouse CD4	Biolegend	100485
APC anti-mouse IFN-γ	Biolegend	505810
PE-Dazzle 594 anti-mouse IL-10	Biolegend	505034
PerCP/Cyanine5.5 anti-mouse/human CD44	Biolegend	103032
Alexa Fluor® 700 anti-mouse CD62L	Biolegend	104426
BD Horizon™ BUV395 Rat Anti-Mouse CD45	BD Biosciences	564279
